# Monoclonal antibodies that target fibroblast growth factor receptor 2 isoform b and Claudin‐18 isoform 2 splicing variants in gastric cancer and other solid tumours

**DOI:** 10.1002/ctm2.1736

**Published:** 2024-06-14

**Authors:** Masuko Katoh, Izuma Nakayama, Zev A Wainberg, Kohei Shitara, Masaru Katoh

**Affiliations:** ^1^ M & M Precision Medicine Tokyo Japan; ^2^ Department of Gastroenterology and Gastrointestinal Oncology National Cancer Center Hospital East Kashiwa Japan; ^3^ Department of Medicine University of California Los Angeles Los Angeles California USA; ^4^ Department of Omics Network National Cancer Center Tokyo Japan

Splicing variants are generated from multiexon genes due to alternative splicing of pre‐mRNAs through intronic removal and exon connection depending on cis‐acting nucleotide sequences (splicing enhancers and silencers) and trans‐acting splicing regulators (ESRP1, ESRP2, SF3B1, SRSF2, U2AF1 and others).^1^, ^2^ Splicing variants are also generated using alternative promoters adjacent to alternative first exons. Because of the proteomic and transcriptional diversity in splicing variants, a variety of cellular processes, such as apoptosis/survival, drug sensitivity/resistance, epithelial/mesenchymal plasticity, immune surveillance/tolerance, senescence/proliferation and vascular homeostasis, are regulated by isoform switching.

Treatments targeting splicing variants are largely classified into (i) biologics or chimeric antigen receptor T (CAR‐T) cells, which target splicing isoforms that are preferentially overexpressed in tumour cells (Figure 1), such as fibroblast growth factor receptor 2 isoform b (FGFR2b)^3^ and Claudin‐18 isoform 2 (CLDN18.2)^4^; (ii) small‐molecule compounds, which target splicing regulators, including SF3B1 inhibitors (E7107 and H3B‐8800); and (iii) antisense oligonucleotides (ASOs), which target splice site mutations or interfaces between pre‐mRNAs and splicing regulators. Small‐molecule compounds and ASOs in the field of alternative splicing will be reviewed elsewhere.^1^, ^2^ Here, recent progress on splicing isoform‐targeted biologics for the treatment of patients with gastric or gastroesophageal junction adenocarcinoma (GEA) in the later stage of clinical development will be reviewed (Table 1), and future perspectives will be discussed.

**FIGURE 1:**
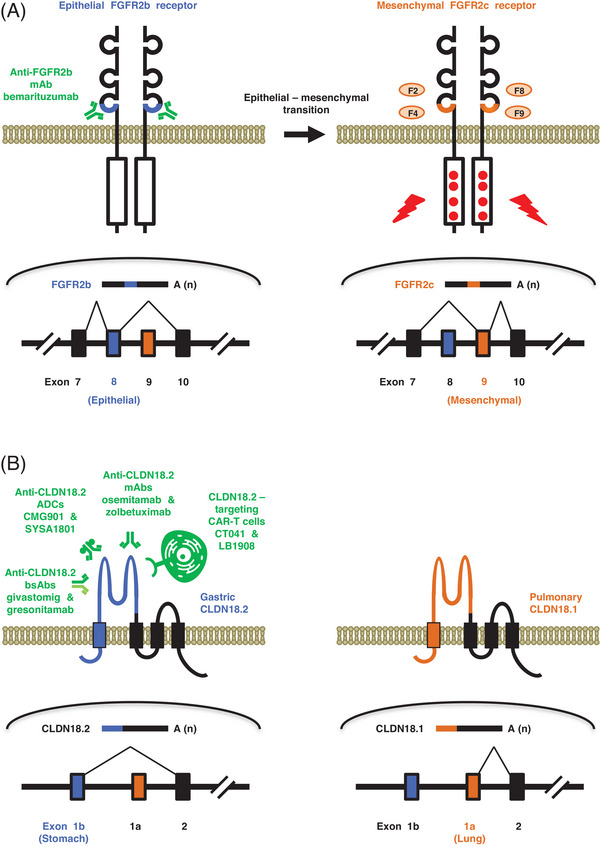
Fibroblast growth factor receptor 2 isoform b (FGFR2b) and Claudin‐18 isoform 2 (CLDN18.2) splicing variants and targeted therapy. (A) FGFR2b and FGFR2c splicing variants. Epithelial FGFR2b and mesenchymal FGFR2c are generated via alternative splicing of exclusive exons. FGFR2b and FGFR2c are receptor tyrosine kinase transducing signals of FGF ligands: FGFR2b for FGF7, FGF10 and FGF22; and FGFR2c for FGF2 (F2), FGF4 (F4), FGF8 (F8) and FGF9 (F9). FGFR2b is overexpressed as a result of gene amplification in patients with gastric or gastroesophageal junction adenocarcinoma (GEA) and other cancers. Bemarituzumab is an anti‐FGFR2b monoclonal antibody (mAb) that is in the later phase of clinical development. (B) CLDN18.1 and CLDN18.2 splicing variants. Pulmonary CLDN18.1 and gastric CLDN18.2 are generated using alternative promoters. CLDN18.1 and CLDN18.2 are tight‐junction four‐transmembrane proteins that function as physiological barriers for aeroantigens and gastric acid, respectively. CLDN18.2 is overexpressed in patients with GEA, colorectal cancer, pancreatic cancer and ovarian cancer. Anti‐CLDN18.2 mAbs (osemitamab, zolbetuximab and others), CLDN18.2‐targeting antibody‐drug conjugates (ADCs; CMG901, SYSA1801 and others), CLDN18.2‐targeting bispecific antibodies (bsAbs; givastomig, gresonitamab and others) and CLDN18.2‐targeting CAR‐T cells (CT041, LB1908 and others) are in clinical development for patients with CLDN18.2‐overexpressing tumours.

**TABLE 1:** Clinical trials of splicing isoform‐targeting monoclonal antibodies (mAbs) for gastroesophageal junction adenocarcinoma (GEA) patients.

Treatment	Clinical trial	Patients	Clinical activity
Bemarituzumab monotherapy	P1 (FPA144‐001, NCT02318329)	GEA (*n* = 56) & other solid tumours (*n* = 23)	ORR, 18% (5/28) in FGFR2b+ GEA ^5^
Bemarituzumab or placebo plus mFOLFOX6	P2 (FIGHT, NCT03694522)	FGFR2b+ GEA, bemarituzumab‐mFOLFOX6 (*n* = 77) versus placebo‐mFOLFOX6 (*n* = 78)	Bemarituzumab versus placebo:
			ORR, 48.1% versus 33.3%;
			mPFS, 9.5 versus 7.4 months
			(HR 0.72; 95% CI 0.49–1.08);
			mOS, 19.2 versus 13.5 months
			(HR 0.77; 95% CI 0.52–1.14)^6^
Bemarituzumab or placebo plus mFOLFOX6	P3 (FORTITUDE‐101, NCT05052801)	1st‐line FGFR2b+ GEA, bemarituzumab‐mFOLFOX6 versus placebo‐mFOLFOX6	NR (Estimated completion in August 2025)
Bemarituzumab or placebo plus mFOLFOX6/ICI	P3 (FORTITUDE‐102, NCT05111626)	1st‐line FGFR2b+ GEA, bemarituzumab‐mFOLFOX6/ICI versus placebo‐mFOLFOX6/ICI	NR (Estimated completion in September 2026)
Zolbetuximab monotherapy	P2a (MONO, NCT01197885)	2nd‐ or more‐line CLDN18.2+ GEA (*n* = 54)	ORR, 9% (4/43)^4^
Zolbetuximab or placebo plus mFOLFOX6	P3 (SPOTLIGHT, NCT03504397)	1st‐line CLDN18.2+ GEA, zolbetuximab‐mFOLFOX6 (*n* = 283) versus placebo‐mFOLFOX6 (*n* = 282)	Zolbetuximab versus placebo,
			ORR, 48% versus 48%;
			mPFS, 10.6 versus 8.7 months
			(HR 0,75; 95% CI 0.60‐0.94)
			mOS, 18.2 versus 15.5 months
			(HR 0.75; 95% CI 0.60–0.94)^4^
Zolbetuximab or placebo plus CAPOX	P3 (GLOW, NCT03653507)	1st‐line CLDN18.2+ GEA, zolbetuximab‐CAPOX (*n* = 254) versus placebo‐CAPOX (*n* = 253)	Zolbetuximab versus placebo,
			ORR, 53.8% versus 48.8%;
			mPFS, 8.2 versus 6.8 months
			(HR 0,69; 95% CI 0.54–0.87)
			mOS, 14.4 versus 12.2 months
			(HR 0.77; 95% CI 0.62–0.97)^4^

Abbreviations: bemarituzumab, anti‐FGFR2b human mAb; CAPOX, capecitabine and oxaliplatin chemotherapy; CI, confidence interval; CLDN18.2+, CLDN18.2 overexpression by immunohistochemistry (3+/2+ membranous staining, the requirement for CLDN18.2+ tumour cell proportion depends on a clinical trial); FGFR2b+, FGFR2b overexpression by immunohistochemistry (3+ or 3+/2+ membranous staining) and/or *FGFR2* amplification by fluorescence in situ hybridization or circulating tumour DNA assay (definition of FGFR2b+ depends on a clinical trial); GEA, gastric or gastroesophageal junction adenocarcinoma; HR, hazard ratio; ICI, immune checkpoint inhibitor; mAb, monoclonal antibody; mFOLFOX6, modified 5‐fluorouracil, leucovorin and oxaliplatin chemotherapy; mOS, median overall survival; mPFS. median progression‐free survival; NR, not reported; ORR, objective response rate; zolbetuximab, anti‐CLDN18.2 mouse/human chimeric mAb.

## 1 BEMARITUZUMAB FOR GEA PATIENTS

Bemarituzumab, also known as FPA144, is a humanized anti‐FGFR2b monoclonal antibody (mAb) that likely exerts its effects through FGF7/10/22‐FGFR2b signalling blockade and antibody‐dependent cellular cytotoxicity and enhances natural killer cell‐mediated antitumour immunity.^3^ Bemarituzumab monotherapy in the phase I FPA144‐001 study (NCT02318329) revealed an objective response rate (ORR) of 18% in GEA patients with FGFR2b amplification.^5^

In the randomized, double‐blinded phase II FIGHT study (NCT03694522), the combination of bemarituzumab plus FOLFOX showed improved clinical activity in comparison with placebo plus FOLFOX in GEA patients with FGFR2b overexpression or *FGFR2* amplification: the median progression‐free survival was 9.5 versus 7.4 months (hazard ratio [HR] 0.72; 95% confidence interval [CI] 0.49–1.08); and the median overall survival was 19.2 versus 13.5 months (HR 0.77; 95% CI 0.52–1.14).^6^

Bemarituzumab was granted a breakthrough therapy designation by the US Food and Drug Administration (FDA); the phase III FORTITUDE‐101 study of bemarituzumab plus chemotherapy (NCT05052801) and a phase III FORTITUDE‐102 study of bemarituzumab plus chemotherapy and nivolumab (NCT05111626) for the first‐line treatment of GEA patients harbouring FGFR2b overexpression are ongoing (Table 1).^3^

## 2 ZOLBETUXIMAB FOR GEA PATIENTS

Zolbetuximab, also known as IMAB362, is a chimeric anti‐CLDN18.2 mAb that induces antibody‐dependent cellular cytotoxicity and complement‐dependent cytotoxicity towards CLDN18.2+ tumour cells.^4^

Zolbetuximab monotherapy achieved an ORR of 9% in GEA patients with CLDN18.2 overexpression in a phase IIa MONO study (NCT01197885), and compared with placebo plus chemotherapy, zolbetuximab plus chemotherapy improved clinical outcomes in the SPOTLIGHT (NCT03504397) and GLOW (NCT03653507) phase III studies (Table 1).

Zolbetuximab was approved for the treatment of CLDN 18.2‐positive GEA in Japan on March 26. However, the FDA's complete response letter pointed out unresolved deficiencies in a third‐party manufacturing facility.^7^ Reapplication of zolbetuximab to the FDA has not yet been announced as of 23 May 2024.

## 3 EPITOPE‐LOSING ISOFORM SWITCH

Loss of epitopes or targeted proteins is a common mechanism of resistance to antibody‐based biologics and CAR‐T cells.^8^ Human epidermal growth factor receptor 2 (HER2) isoform switches from epitope‐positive to epitope‐negative splicing variants elicited resistance to the anti‐HER2 mAb trastuzumab in breast cancer patients, while CD19 and CD22 isoform switches into epitope‐negative splicing variants, and CD22 isoform switch into a noncoding splicing variant, caused resistance to CAR‐T cells in B‐lymphoid malignancies.^1^, ^2^

The FGFR2b isoform is generated by ESRP splicing regulators in tumour cells with an epithelial phenotype, whereas the FGFR2c isoform is induced in tumour cells with a mesenchymal phenotype as a result of ESRP repression.^9^ FGFR2 isoform switching from FGFR2b to FGFR2c due to epithelial–mesenchymal transition is associated with cancer aggressiveness and advanced clinical stages^3^ and might limit the clinical benefits of bemarituzumab.

The CLDN18.1 and CLDN18.2 isoforms are transcribed by pulmonary and gastric promoters, respectively.^10^ However, in companion diagnostics of zolbetuximab, immunohistochemistry using a clone 43‐14A antibody detected both the CLDN18.1 and CLDN18.2 isoforms because they bound to a common intercellular region.^4^ Consequently, potential CLDN18 isoform switching to CLDN18.1 owing to gastric‐to‐pulmonary transdifferentiation or downregulated CLDN18.2 expression owing to gastric‐to‐intestinal transdifferentiation in GEA patients treated with zolbetuximab remains to be investigated.

## 4 BEYOND MAB DRUGS

MAB‐derived biologics, such as antibody‐drug conjugates, bispecific antibodies, radioimmunoconjugates and CAR‐T cells are options for enhancing the clinical effectiveness of mAb drugs.^3^, ^4^ The clinical development of human/humanized anti‐CLDN18.2 mAbs with enhanced antitumour activity (ASKB589, MIL93, osemitamab and ZL‐1211), CLDN18.2‐targeting antibody‒drug conjugates with monomethyl auristatin E payload (CMG901 and SYSA1801), CLDN18.2‐targeting bispecific antibodies (givastomig, gresonitamab, PT886 and Q‐1802 bind to 4‐1BB, CD3, CD47 and programmed death ligand 1, respectively) and CLDN18.2‐targeting CAR‐T cells (CT041, CT048, LB1908 and RD07) for the treatment of CLDN18.2+ cancer patients is ongoing.^4^

## 5 BEYOND GEA PATIENTS

Immunohistochemical analyses are applied as companion diagnostics with the inclusion criteria of 3+/2+ FGFR2b membranous staining in ≥ 10% of tumour cells for bemarituzumab (NCT05052801 and NCT05111626) and 3+/2+ CLDN18.1 or CLDN18.2 membranous staining in ≥ 75% of tumour cells for zolbetuximab (NCT03504397 and NCT03653507).

Because FGFR2b and CLDN18.2 are also overexpressed in non‐GEA tumours, a phase I/II clinical trial of bemarituzumab monotherapy for patients with FGFR2b+ solid tumours, such as intrahepatic cholangiocarcinoma, lung adenocarcinoma, ovarian epithelial carcinoma and triple‐negative breast cancer, is ongoing, with an estimated study completion date in July 2027 (FORTITUDE‐301 study, NCT05325866), while a phase II clinical trial of zolbetuximab plus chemotherapy for first‐line treatment of patients with CLDN18.2+ metastatic pancreatic adenocarcinoma is ongoing, with an estimated completion date in April 2025 (NCT03816163).

## 6 CONCLUSION

Bemarituzumab and zolbetuximab are the first‐in‐human biologics that target the FGFR2b and CLDN18.2 splicing variants, respectively, and are expected to reduce adverse events caused by FGFR2c and CLDN18.1 targeting. Bemarituzumab and zolbetuximab had single‐agent ORRs of approximately 10%–20% and had significant clinical benefits when combined with chemotherapy. Clinical trials aimed at expanding the indications of bemarituzumab and zolbetuximab and those of other biologics and CAR‐T cells targeting CLDN18.2+ tumours are ongoing.

## AUTHOR CONTRIBUTIONS

Masuko Katoh and Masaru Katoh prepared the initial manuscript. Izuma Nakayama, Kohei Shitara and Zev A Wainberg discussed and revised the manuscript.

## CONFLICT OF INTEREST STATEMENT

Masaru Katoh, Masuko Katoh and Izuma Nakayama declare no conflict of interest associated with this manuscript. Kohei Shitara has acted as a consultant and/or adviser of ALX Oncology, Amgen, Astellas, AstraZeneca, Bayer, Boehringer Ingelheim, Bristol Myers Squibb, Daiichi Sankyo, Guardant Health Japan, Janssen, Merck Pharmaceutical, Novartis, Ono Pharmaceutical, Takeda and Zymeworks Biopharmaceuticals, and has received research funding from Astellas, Amgen, Chugai, Daiichi Sankyo, Eisai, Merck Pharmaceutical, Ono Pharmaceutical, PRA Health Sciences, Syneos Health and Taiho Pharmaceutical. Zev A. Wainberg has been a consultant for Amgen, Arcus, Astellas, Astra Zeneca, Bayer, Bristol Myers Squibb, Daiichi Sankyo, Gilead, Ipsen, Merck, Novartis and Seagen, has received grants from Arcus, Bristol Myers Squibb and Plexxikon, and has served on the data and safety monitoring board for Compass, Daiichi Sankyo, Mirati and Pfizer.
